# Exploring the Links Between Self-Compassion, Body Dissatisfaction, and Acceptance of Cosmetic Surgery in Young Italian Women

**DOI:** 10.3389/fpsyg.2019.02698

**Published:** 2019-12-03

**Authors:** Amanda Nerini, Camilla Matera, Cristian Di Gesto, Giulia Rosa Policardo, Cristina Stefanile

**Affiliations:** ^1^Department of Education, Languages, Intercultures, Literatures and Psychology, University of Florence, Florence, Italy; ^2^Department of Health Sciences, University of Florence, Florence, Italy

**Keywords:** self-compassion, body dissatisfaction, cosmetic surgery, physical appearance comparison, internalization

## Abstract

This study aimed to examine the association between positive (self-kindness, common humanity, and mindfulness) and negative (isolation, self-judgment, and over-identification) components of self-compassion, and both body dissatisfaction and acceptance of cosmetic surgery among women, through the mediation (for the negative components) of internalization and physical appearance comparison. The participants were 220 young Italian women aged 19–31 (*M* = 21) years, who completed a questionnaire assessing the variables of interest. Path analysis indicated that higher mindfulness was directly linked to lower acceptance of cosmetic surgery. Mindfulness presented the strongest link with cosmetic surgery, as it was directly associated with acceptance of cosmetic surgery for both social and interpersonal motivations and with consideration of undergoing some cosmetic procedures. Common humanity and self-kindness were related to acceptance of cosmetic surgery for social reasons. Over-identification seemed to be associated with body dissatisfaction and acceptance of cosmetic surgery both directly and indirectly through internalization and physical appearance comparison. Self-judgment and isolation did not present a significant association with either body dissatisfaction or acceptance of cosmetic surgery. These findings confirm that psychological assessment of women who are interested in cosmetic surgery is highly recommended. Interventions should not consider self-compassion as a whole, but they should rather focus on some of its components. The role of over-identification seems to be especially pivotal, as higher scores on this dimension are linked to higher levels of body dissatisfaction and greater acceptance of cosmetic surgery.

## Introduction

Cosmetic surgery is an optional, or medically unnecessary, procedure (Diaz, 2012) requested by a patient to correct imperfections and improve appearance (Barone et al., 2016). Some studies have shown a positive link between body dissatisfaction and acceptance of cosmetic surgery among women, suggesting that people may consider cosmetic surgery as a means to obtain both intrapsychic benefits (e.g., higher self-esteem) and social rewards deriving from appearing more attractive to others (Markey and Markey, 2009; Slevec and Tiggemann, 2010; Menzel et al., 2011; Lunde, 2013). Nevertheless, some experimental evidence suggests that body image quality of life and self-esteem do not increase in women who have undergone some cosmetic surgical procedures, even though their body dissatisfaction can decrease (Sobanko et al., 2018).

Italy ranks sixth in the list of countries with the largest number of women who underwent cosmetic surgical procedures in 2016 (International Society of Aesthetic Plastic Surgery, 2017). Such an interest in cosmetic practices can derive from the emphasis that the Italian society places on physical appearance (Dakanalis et al., 2015; Barcaccia et al., 2018). A study conducted by Mondini et al. (1996) revealed that cosmetic surgery was already present in the Italian mass media two decades ago. It was typically presented as an effective manipulation strategy that might help women to achieve common aesthetic standards that are difficult to be reached with natural methods.

What factors might be associated with women’s consideration of cosmetic surgery? Through the present study, we aimed to examine if an attitude of kindness and understanding toward oneself in the face of inadequacies, failures, and personal difficulties—which is called self-compassion (Neff, 2003)—can be significantly associated with lower body dissatisfaction and lower acceptance of cosmetic surgery among women. Indeed, self-compassion was found to mitigate the maladaptive outcomes of poor body image (Braun et al., 2016).

Self-compassion enhances one’s ability to respond to threats and environmental stressors (such as pressures related to one’s physical appearance) in a nonreactive and nonjudgmental manner (Ferreira et al., 2013). This is due to a lower tendency to self-criticize, which may reduce the extent to which people perceive negative thoughts and feelings as severe, making them more easily acceptable (Neff, 2003). Self-compassion entails three positive (self-kindness, common humanity, and mindfulness) and three negative components (self-judgment, isolation, and over-identification) (Neff, 2016). According to Neff (2003), self-kindness refers to an attitude of care and understanding toward oneself in the face of suffering. Common humanity allows one to perceive and classify personal experiences of failure as elements shared by humanity. Mindfulness is a state of awareness, attention, and acceptance of one’s negative thoughts and feelings in a balanced and nonjudgmental way. Self-judgment is the tendency to disapprove and harshly judge one’s flaws and inadequacies. Isolation, which is the perception of being the only one suffering or making mistakes, occurs when people tend to feel separate and cutoff from the rest of the world while thinking about their own inadequacies. Over-identification is the excessive identification with and absorption in one’s feelings and emotions, and it involves the tendency to obsess and fixate on everything that is wrong. High levels of self-compassion can be therefore expressed in terms of high self-kindness, common humanity, and mindfulness, and low self-judgment, isolation, and over-identification.

The positive and negative components do not seem completely symmetrical. Much research (Gilbert, 2005; Smith and Zautra, 2008; Longe et al., 2010; Costa et al., 2016; Brenner et al., 2017) has suggested that the positive components combine into one factor, whereas the negative components combine into another one (called self-criticism). Distinct processes and internal systems seem to be activated by these two factors (Brenner et al., 2017). The former could be considered a resilience factor (Neff and McGehee, 2010), which might allow people to relax and engage in behaviors that foster health and well-being (Gilbert, 2005), whereas self-criticism might represent a vulnerability factor (Dunkley et al., 2009), which could make people more severe toward their own failures and inadequacies (Gilbert et al., 2011). Notably, Thompson and Zuroff (2004) identified two distinct forms of self-criticism, which are comparative and internalized self-criticism. Comparative self-criticism is a negative view of the self in comparison with others, whereas internalized self-criticism is a negative view of the self in comparison with internal standards, which are often high and hardly achievable. According to this perspective, the negative components of self-compassion seem to be highly related to internalization and physical appearance comparison, which are two key processes responsible for body dissatisfaction and acceptance of cosmetic surgery (Keery et al., 2004; Stefanile et al., 2009; Menzel et al., 2011; Rodgers et al., 2011; Nerini, 2015; Nerini et al., 2016). The internalization of social ideals can be defined as the internal incorporation of societal standards of attractiveness to the point that these values become guiding principles (Thompson et al., 2004). Physical appearance comparison is the tendency to evaluate dimensions of the self, such as body image, through comparison with others (Thompson et al., 1991). Women who internalize beauty ideals may be more likely to engage in physical appearance comparison to establish if they meet shared cultural standards of beauty (Clay et al., 2005; Durkin et al., 2007; Matera et al., 2013a). Internalization and physical appearance comparison could therefore mediate the relationship between the negative components of self-compassion (self-criticism) and both body dissatisfaction and acceptance of cosmetic surgery.

Most studies on the association between self-compassion and body dissatisfaction treated self-compassion as a global construct, neglecting its multidimensional nature. To the best of our knowledge, the only study that examined the relationship between all the dimensions that comprise self-compassion and body image is the one by Wasylkiw et al. (2012). The authors found self-judgment to be the only significant predictor of body preoccupation in undergraduate women. The few additional studies that viewed self-compassion as a multidimensional construct examined the role of only some of its components (Dijkstra and Barelds, 2011; Webb and Forman, 2013; Ferreira et al., 2014; Rodgers et al., 2017).

Only two studies examined the relationship between self-compassion and acceptance of cosmetic surgery, examining only a single subcomponent of self-compassion (mindfulness). Manshadi et al. (2014) investigated body satisfaction and mindfulness in cosmetic surgery candidates in comparison with people who have not undergone cosmetic surgical procedures. Cosmetic surgery patients had less positive body satisfaction and lower levels of mindfulness. Naami and Salehi (2016) showed a significant negative relationship between mindfulness and acceptance of cosmetic surgery in high school students.

## The Present Study

The present study aimed to test a model in which self-compassion, conceived as a multidimensional construct, was associated with body dissatisfaction and acceptance of cosmetic surgery *via* internalization and physical appearance comparison. We predicted that the positive components (self-kindness, common humanity, and mindfulness) would be directly associated with body dissatisfaction and acceptance of cosmetic surgery. The more women can respond to threats or environmental stressors in a nonreactive and nonjudgmental manner, the more favorably they might look at their bodies. Such a mental state may be associated with lower dissatisfaction with one’s body and lower acceptance of cosmetic surgery as an appearance enhancement strategy (Hypothesis 1). Based on previous research findings (Thompson and Zuroff, 2004), the negative components were hypothesized to be related to both body dissatisfaction and acceptance of cosmetic surgery by fostering both internalization of societal standards of beauty and comparisons with the appearance of others (Hypothesis 2). We also posited a link from internalization to physical appearance comparison (Nerini et al., 2014; Matera et al., 2015; Stefanile et al., 2015), and from body dissatisfaction to acceptance of cosmetic surgery (Markey and Markey, 2009; Slevec and Tiggemann, 2010; Menzel et al., 2011; Lunde, 2013). Body mass index (BMI) was included to control for its effect, and the components of self-compassion were allowed to covary.

## Methods

### Participants

The participants included 220 Caucasian Italian university women aged 19–31 years (*M* = 21, SD = 1.88). The mean BMI of the sample was 21.02 (SD = 3.17), ranging between 14.2 and 35.2. Most of the participants (82.7%) lived in central Italy, 10% in northern Italy, and 6.8% in southern Italy or on islands. Most of them (94.5%) reported being unmarried, whereas 5.5% reported being married or cohabiting. Regarding education, 91.3% of them had high school diplomas, 7.8% had bachelor’s degrees, and 0.9% had master’s degrees. Most of the participants (96.3%) defined themselves as students, whereas 3.7% defined themselves as workers (1.8% were occasional employees, 0.9% part-time employees, 0.5% full-time employees, and 0.5% were looking for a first job). In terms of sexual orientation, 94.5% of them reported being heterosexual.

### Measures

#### Self-Compassion

The Italian version (Petrocchi et al., 2013) of the Self-Compassion Scale (Neff, 2003) is a 26-item scale that was used to measure the six components of self-compassion along a five-point Likert scale (1 = almost never; 5 = almost always). Self-kindness refers to one’s ability to be caring and understanding with oneself (four items, e.g., “When I’m going through a very hard time, I give myself the caring and tenderness I need”; alpha = 0.83). Self-judgment refers to one’s tendency to be harshly self-critical (five items, e.g., “When I see aspects of myself that I don’t like, I get down on myself”; alpha = 0.88). Common humanity entails the ability to remind oneself that suffering is part of human nature (five items, e.g., “When I feel inadequate in some way, I try to remind myself that feelings of inadequacy are shared by most people”; alpha = 0.77). Isolation is the lack of awareness that all human beings experience suffering and failure (four items, e.g., “When I fail at something that’s important to me, I tend to feel alone in my failure”; alpha = 0.84). Mindfulness involves awareness of, attention to, and acceptance of one’s painful experiences in a balanced and nonjudgmental way (four items, e.g., “When I fail at something important to me, I try to keep things in perspective”; alpha = 0.74). Over-identification refers to the excessive identification with and absorption in one’s feelings and emotions (four items, e.g., “When I’m feeling down, I tend to obsess and fixate on everything that’s wrong”; alpha = 0.77). Higher scores on self-kindness, common humanity, and mindfulness, and lower scores on self-judgment, isolation, and over-identification represented higher levels of self-compassion.

#### Internalization

The internalization-general subscale of the Italian version (Stefanile et al., 2019) of the Sociocultural Attitudes Toward Appearance Questionnaire-4 Revised (Schaefer et al., 2017) was used to assess the degree to which one has internalized sociocultural ideals regarding beauty. This 10-item subscale (e.g., “It is important to me to be attractive”) ranges from 1 (definitively disagree) to 5 (definitively agree). Higher scores represented greater levels of internalization (alpha = 0.86).

#### Physical Appearance Comparison

We adopted an Italian version (Stefanile et al., 2010) of the Physical Appearance Comparison Scale (Thompson et al., 1991) to assess the level to which people make social comparisons related to their appearance. This four-item scale (e.g., “In social situations, I sometimes compare my figure to the figures of other people”) ranges from 1 (never) to 5 (always). Higher scores represented greater levels of physical appearance comparisons (alpha = 0.86).

#### Body Dissatisfaction

We used the Italian version (Matera et al., 2013b) of the Body Shape Questionnaire-14 (Dowson and Henderson, 2001) to assess body dissatisfaction. The scale has 14 items (e.g., “I felt ashamed of my body”) rated along a six-point Likert scale (1 = never; 6 = always). The questionnaire asks the participants to respond on the basis of the past 2 weeks prior to administration. High scores indicated greater levels of general body dissatisfaction (alpha = 0.95).

#### Acceptance of Cosmetic Surgery

We measured acceptance of cosmetic surgery with the Italian version (Stefanile et al., 2014) of the Acceptance of Cosmetic Surgery Scale (Henderson-King and Henderson-King, 2005). This scale has 15 items with a three-factor structure: the Intrapersonal subscale measures attitudes related to the self-oriented benefits of cosmetic surgery (five items; e.g., “Cosmetic surgery can be a big benefit to people’s self-image”; alpha = 0.93), the Social subscale assesses social motivations for having cosmetic surgery (five items; e.g., “If it would benefit my career, I would think about having plastic surgery”; alpha = 0.83), and the Consider subscale measures the probability that a participant would consider having cosmetic surgery (five items; e.g., “In the future, I could end up having some kind of cosmetic surgery”; alpha = 0.92). The scale ranges from 1 (definitively disagree) to 7 (definitively agree). High scores indicated high levels of acceptance of cosmetic surgery.

#### Sociodemographic Details and Body Mass Index

Each participant reported her age, sex, sexual orientation, race, educational level, occupational status, and relationship status. We calculated BMIs (kg/m^2^) using the participants’ reported weights and heights.

### Procedure

Using opportunistic sampling techniques, we recruited the study participants from the School of Psychology at the university with which the authors were affiliated. During regular undergraduate and graduate classes, we asked the students to take part in a study on body image. Participation in the study was voluntary, and we did not provide incentives to the participants. To be eligible for the study, the participants were needed to be 18 years or older women. We obtained informed consent from each participant prior to administering the questionnaire. Participants completed measures in paper-and-pencil format. The questionnaire was anonymous, did not ask for any personally identifiable information, and took about 30 min to complete. The Ethical Committee of the University of Florence approved the study procedures.

### Data Analysis

We examined the fit of three models, in which the self-compassion dimensions were posited as predictors of the three ACSS subscales respectively (Model 1: Intrapersonal; Model 2: Social; Model 3, Consider); the role of internalization, physical appearance comparison, and body dissatisfaction was considered in each model. Less than 1% of the data was missing. We used a mean imputation process to replace the missing values. All the assumptions for path analysis were satisfied (Streiner, 2005). The hypotheses were tested using Amos (version 22; IBM SPSS, Chicago, IL); we used bootstrapping to test mediation by estimating the presence and size of the indirect (i.e., mediated) effects (Rucker et al., 2011). The sample size in the present study was bigger than the recommended size of 200 participants (Weston and Gore, 2006). We adopted the maximum likelihood procedure to derive the parameter estimates and used the following goodness-of-fit indices: the χ^2^/df ratio, a good score of which is 2 or below; the comparative fit index (CFI); the Tucker-Lewis index (TLI); the incremental fit index (IFI), the value of which should be higher than 0.95; the normed fit index (NFI), a good score of which is more than 0.90; the root mean square error of approximation (RMSEA); a 90% confidence interval for RMSEA (RMSEA 90% CI); and the standardized root mean square residual (SRMR). RMSEA and SRMR are considered acceptable if they are 0.08 or lower (Hooper et al., 2008).

## Results

Table 1 shows the descriptive statistics (means and standard deviations) and the intercorrelations among the variables.

**Table 1:** Means (*M*), standard deviations (SD), and intercorrelations between all variables.

	1	2	3	4	5	6	7	8	9	10	11	12	*M* (SD)
1. Self-kindness	1												2.54 (0.80)
2. Self-judgment	−0.67^***^	1											3.23 (0.92)
3. Common humanity	0.57^***^	−0.32^***^	1										2.83 (0.80)
4. Isolation	−0.50^***^	0.69^***^	−0.33^***^	1									3.00 (0.97)
5. Mindfulness	0.75^***^	−0.52^***^	0.56^***^	−0.56^***^	1								2.91 (0.78)
6. Over-identification	−0.54^***^	0.72^***^	−0.35^***^	0.75^***^	−0.58^***^	1							3.54 (0.87)
7. Social comparison	−0.32^***^	0.45^***^	−0.19^**^	0.49^***^	−0.35^***^	0.51^***^	1						3.03 (0.99)
8. Internalization	−0.24^***^	0.34^***^	−0.23^***^	0.35^***^	−0.30^***^	0.44^***^	0.61^***^	1					3.51 (0.75)
9. Body dissatisfaction	−0.24^***^	0.38^***^	−0.23^***^	0.42^***^	−0.32^***^	0.45^***^	0.54^***^	0.44^***^	1				3.04 (1.34)
10. Social	−0.15^*^	0.28^***^	−0.24^***^	0.36^***^	−0.29^***^	0.33^***^	0.35^***^	0.31^***^	0.31^***^	1			1.77 (1.07)
11. Consider	−0.19^**^	0.27^***^	−0.21^**^	0.32^***^	−0.32^***^	0.28^***^	0.44^***^	0.41^***^	0.36^***^	0.64^***^	1		3.09 (1.87)
12. Intrapersonal	−0.13^*^	0.16^*^	−0.15^*^	0.22^***^	−0.23^***^	0.23^***^	0.31^***^	0.34^***^	0.24^***^	0.49^***^	0.67^***^	1	3.85 (1.60)
13. BMI	−0.05	−0.02	−0.10	0.04	−0.12	0.04	−0.03	−0.07	0.38^***^	−0.06	−0.07	−0.11	21.03 (3.17)

*N = 220*;

* *p < 0.05*;

** *p < 0.01*;

*** *p < 0.001*.

The data are normally distributed (skewness <1.54; kurtosis <4.11), as the skews for all variables are lower than 2 and kurtosis is lower than 7 (West et al., 1995).

**Figure 1:**
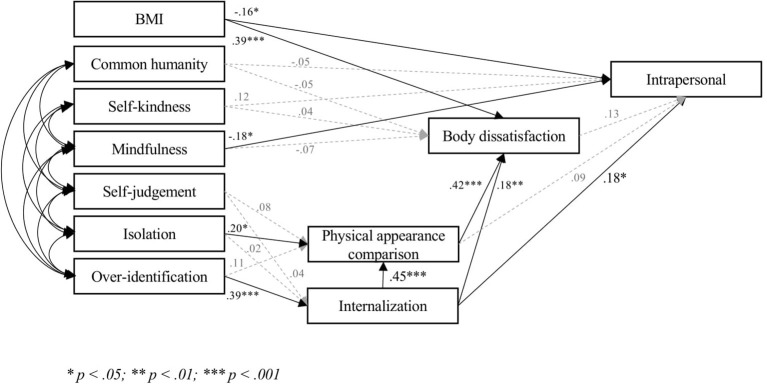
Final model intrapersonal.

**Figure 2:**
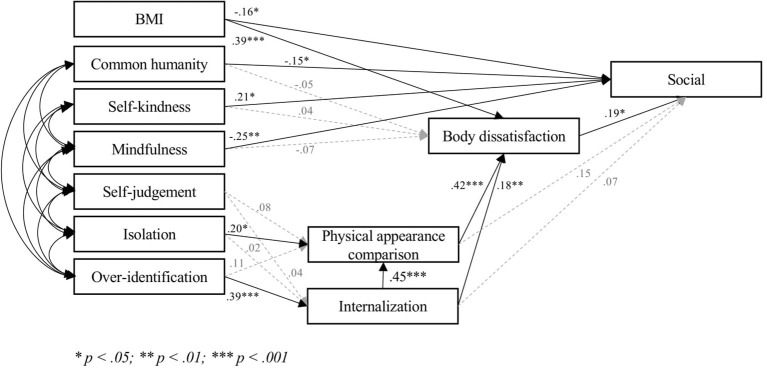
Final model social.

**Figure 3:**
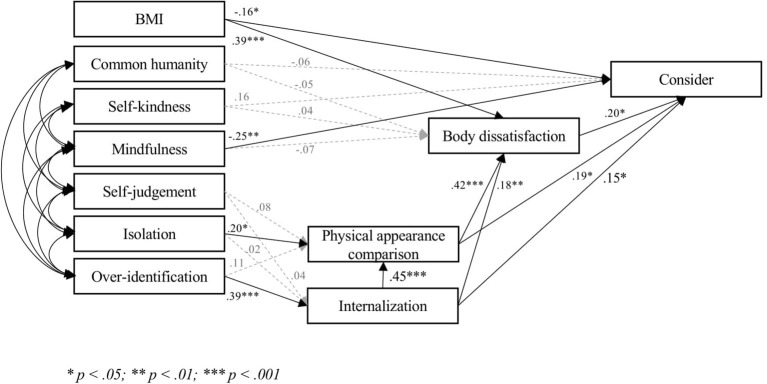
Final model consider.

The three models (Figures 1–3) fitted very well with the data [Model 1, Intrapersonal: *χ*^2^ = 25.25, *p* = 0.19; *χ*^2^/df = 1.26; RMSEA = 0.04 (CI = 0.00; 0.07); SRMR = 0.04; CFI = 0.99; TLI = 0.99; IFI = 0.99; NFI = 0.98: Model 2, Social: *χ*^2^ = 31.30, *p* = 0.05; *χ*^2^/df = 1.56; RMSEA = 0.05 (CI = 0.00; 0.08); SRMR = 0.04; CFI = 0.99; TLI = 0.97; IFI = 0.99; NFI = 0.97. Model 3, Consider: *χ*^2^ = 28.17, *p* = 0.10; *χ*^2^/df = 1.41; RMSEA = 0.04 (CI = 0.00; 0.08); SRMR = 0.04; CFI = 0.99; TLI = 0.98; IFI = 0.99; NFI = 0.98]. Covariances ranged between 0.23 (*p* < 0.001) and 0.63 (*p* < 0.001).

Hypothesis 1 was partially confirmed. None of the positive components were significantly related to body dissatisfaction, whereas mindfulness was significantly related to acceptance of cosmetic surgery in each model. Self-kindness and common humanity were significantly associated with acceptance of cosmetic surgery for social reasons (Model 2), but not with either acceptance of cosmetic surgery for intrapersonal reasons (Model 1) or consideration of undergoing some cosmetic procedures (Model 3). Notably, the relationship between self-kindness and acceptance of cosmetic surgery for social reasons (Model 2) was a *negative* one.

In line with Hypothesis 2, the bootstrapping procedure (Preacher and Hayes, 2008) showed that the indirect effect of over-identification on body dissatisfaction through internalization and physical appearance comparison was significant in the three models (0.188; 95% CI: 0.082; 0.296). Over-identification was related to acceptance of cosmetic surgery for intrapersonal reasons only *via* internalization (0.121; 95% CI: 0.048; 0.236), and was related to both acceptance of cosmetic surgery for social reasons and consideration of cosmetic surgery through internalization, physical appearance comparison, and body dissatisfaction (Social: 0.106; 95% CI: 0.049; 0.204; Consider: 149; 95% CI: 0.068; 0.259).

Isolation was associated with physical appearance comparison, but not with internalization. Contrary to Hypothesis 2, the indirect effect of isolation on body dissatisfaction (0.091; 95% CI: −0.012; 0.207), acceptance of cosmetic surgery for social reasons (0.051; 95% CI: −0.009; 0.122), or consideration of cosmetic surgery (0.062; 95% CI: −0.016; 0.157) was not significant. Isolation was not indirectly related to acceptance of cosmetic surgery for intrapersonal reasons, as the latter was not associated with either physical appearance comparison or body dissatisfaction. Self-judgment was not significantly related to any other variable.

The three models accounted for much of the variance in body dissatisfaction (48%) and for a satisfactory percentage of the variance of acceptance of cosmetic surgery (Intrapersonal: 17%; Social: 22%; Consider: 29%).

## Discussion

Our findings showed that the positive and negative components of self-compassion were not completely symmetrical. Mindfulness presented the strongest link with cosmetic surgery, as it was directly associated with acceptance of cosmetic surgery for both social and interpersonal motivations and with consideration of undergoing some cosmetic procedures. Mindfulness may provide greater distress tolerance (Schoenefeld and Webb, 2013; Webb and Forman, 2013) and higher cognitive and emotional flexibility, making people less interested in cosmetic surgery to enhance their appearance (Manshadi et al., 2014; Naami and Salehi, 2016). Common humanity and self-kindness were related to acceptance of cosmetic surgery for social reasons. Women who reminded themselves that suffering is part of human nature (i.e., common humanity) were less likely to evaluate cosmetic surgery as a means of appearing more attractive to others and gaining social rewards. Surprisingly, an attitude of care and understanding toward oneself (i.e., self-kindness) was associated with *higher* acceptance of cosmetic surgery for social reasons. Even if the simple correlation between the two variables was negative, when all the components of self-compassion were accounted for, the sign of this relationship changed. Probably, the simple correlation and the overlap among the dimensions of self-compassion did not permit to examine the unique effect of self-kindness; only after controlling for the shared variance among all the components, the unique effect of self-kindness could be disentangled. Women who can support themselves kindly seem to be more likely to view cosmetic surgery as a reasonable way to provide themselves satisfaction when they experience difficulties and sorrow in social contexts or during social interactions. Cosmetic surgery might be conceived as a way to take care of oneself in order to obtain external rewards, such as positive feedback and material advantages. Indeed, many people nowadays choose cosmetic surgery as a generous life-changing gift (American Society of Plastic Surgeons, 2017). Contrary to Hypothesis 1, none of the positive components was associated with body dissatisfaction.

In accord with Hypothesis 2, the excessive identification with and absorption in one’s feelings and emotions (i.e., over-identification) seemed to be associated with the likelihood of internalizing sociocultural standards of appearance. This process, in turn, might increase body dissatisfaction and acceptance of cosmetic surgery both directly and indirectly through physical appearance comparison, which could help establish if one is effectively meeting internalized sociocultural standards of beauty (Clay et al., 2005; Durkin et al., 2007; Matera et al., 2013a); if physical appearance comparison discloses that a woman does not match the size and shape of other people, that woman may experience body dissatisfaction.

In our models, self-judgment did not present a significant association with either body dissatisfaction or acceptance of cosmetic surgery. It seems that it is not the tendency to disapprove and harshly judge one’s flaws and inadequacies, but the excessive identification with and absorption in these feelings of inadequacies, that may be relevant for women’s body dissatisfaction. In addition, isolation was not associated with either body dissatisfaction or acceptance of cosmetic surgery, although it was significantly related to physical appearance comparison. Women who felt separate and cutoff from the rest of the world probably felt more depressed and hopeless, and were more likely to compare their appearance with that of others with the aim of verifying their status and feeling less isolated. In other words, in this case physical appearance comparison might not have the aim of restoring one’s body image, but it could be performed in order to improve one’s perception of being connected to others.

This study has some limitations. First, because of the correlational nature of this research, we cannot make causal inferences. Second, we assessed acceptance, but not effective engagement, of cosmetic surgery. Perspective and experimental studies must clarify the causal relationship between the variables and must examine the relationship between attitudes and the actual decision to undergo cosmetic surgical procedures. Third, this study is not exhaustive of potential variables that may protect women from body dissatisfaction and consideration of cosmetic surgery. Future studies could examine if public self-awareness, which is related to both self-compassion (Neff and Vonk, 2009) and acceptance of cosmetic surgery (Matera et al., 2015), might be a relevant mediator of the relationship between these two variables. Moreover, we used a convenience sample, so our findings are not generalizable to the entire population.

These findings confirm that psychological assessment of women who are interested in cosmetic surgery is highly recommended (Mulkens et al., 2012; Brunton et al., 2014). Indeed, cosmetic surgery does not necessarily help women to improve their body image (Sarwer, 2018; Sobanko et al., 2018). If women seek cosmetic surgery without changing their attitude toward the self, they will probably report low self-esteem also after undergoing surgery, which could lead them to look for further cosmetic procedures, without ever feeling comfortable with their own body image. Before undergoing cosmetic procedures, surgeons might propose alternative strategies, such as self-compassion trainings, that could help women to change the way they relate to their body. Based on our findings, this kind of training should not consider self-compassion as a whole, but it should rather focus on some of its components. The role of over-identification seems to be especially pivotal, as higher scores on this dimension are linked to higher levels of body dissatisfaction and greater acceptance of cosmetic surgery. To reduce women’s tendency to excessively identify with their feelings and emotions could either dissuade women from undertaking unnecessary surgical interventions or reduce the likelihood that poor body image is experienced even after some cosmetic procedures are effectively undertaken. Higher levels of mindfulness and common humanity could decrease women’s acceptance of cosmetic surgery as well, at least for social reasons, even though these dimensions appeared to be unrelated to women’s satisfaction with their body. The association between self-kindness and acceptance of cosmetic surgery might be further explored before considering the advantages and disadvantages of trainings that specifically focus on this dimension.

## Data Availability Statement

The datasets generated for this study are available on request to the corresponding author.

## Ethics Statement

The studies involving human participants were reviewed and approved by Ethical Committee of the University of Florence (Italy). The patients/participants provided their written informed consent to participate in this study.

## Author Contributions

All authors listed have made a substantial, direct and intellectual contribution to the work, and approved it for publication.

### Conflict of Interest

The authors declare that the research was conducted in the absence of any commercial or financial relationships that could be construed as a potential conflict of interest.
